# Carbon Transfer Decision Model Based on LMDI Method

**DOI:** 10.1155/2022/3970880

**Published:** 2022-03-09

**Authors:** Yawei Qi, Guangping Rao, Lei Zha, Lu Chen, Yuping Niu

**Affiliations:** School of Information Management, Jiangxi University of Finance and Economics, Nanchang 330032, China

## Abstract

Establishing a coordinated governance mechanism for regional carbon emissions is an essential way to achieve carbon peak and carbon neutrality, while the study of interprovincial carbon emissions transfer is one of the important foundations of regional carbon emissions coordinated governance research. Based on the multiregional input-output (MRIO) model, this study calculated the carbon emissions from both the producers' perspective and the consumers' perspective and analyzed the interprovincial net carbon emissions transfer decision. Furthermore, the logarithmic mean Divisia index (LMDI) method was adopted to decompose the factors that affect the province's net carbon emissions into technological effect, structural effect, input-output effect, and scale effect. It was revealed that the input-output effect was the primary influencing factor of the net carbon transfer at the provincial level.

## 1. Introduction

China's economy has grown massively since the 21st century. As of 2020, China has achieved its first centenary goal—building a moderately prosperous society in all respects and is striding forward to its second centenary goal. However, the emission of carbon dioxide (CO_2_) is also rising along with the total economic volume, and China has become the world's largest carbon emitter. The issue of greenhouse gas emissions is getting more and more attention. On March 15, 2021, the Chinese government stated to have carbon emissions peak before 2030 and achieve carbon neutrality by 2060. This is a major decision under the goal of building a community of shared future for all humankind.

China has a vast territory. The differences in resource endowments and climate environment in different regions have not only brought differences in industrial structure between regions but also differences in CO_2_ emissions of regional production activities. During regional trading, the production of goods and the provision of services lead to carbon emissions in production areas, but goods and services are actually consumed by consumption areas; therefore, CO_2_ emissions are transferred along with the flow of goods and services. If the inter-regional transfer of carbon emissions is ignored during the promotion of carbon emissions reduction, on the one hand, it may cause some regions to import high-carbon products from other regions for carbon emissions reduction. It will impair the effectiveness of emissions reduction policy, and even cause partial decrease with overall increase [1, 2]. On the other hand, it may cause high-polluting and high-energy-consuming industries to migrate from high-carbon emissions areas to low-carbon emissions areas. It is not conducive to take advantage of resource endowments in various regions, and neither conducive to promoting the development and utilization of clean energy and encouraging low-carbon consumption and low-carbon lifestyle. Therefore, to advance carbon emissions reduction, we need to think from the perspective of regional synergy. However, before planning how to scientifically promote the fair distribution of CO_2_ reduction between regions, we need to pay attention to the following two issues. First, how is the regional distribution of carbon emissions transfer in China? Second, what are the main factors affecting the transfer of carbon emissions between regions in China? Therefore, this study is committed to carrying out the analysis of interprovincial carbon emission transfer, characterizing the relationship of carbon emission transfer, discussing its influencing factors, and consolidating the foundation for the research on carbon emission collaborative governance.

## 2. Literature Review

With respect to the calculation of carbon emissions transfer, more and more scholars adopted the multiregional input-output (MRIO) model in recent years. The single-region input-output (SRIO) model was first constructed by Leontief [3] and then expanded to multiple regions and widely applied in environmental impact assessment (EIA) [1, 2, 4].

Various scholars have integrated the MRIO model with the actual situation in China. Guo et al. [5], Yao et al. [6], and Zhang et al. [7] used the MRIO model to calculate and analyze China's interprovincial carbon emissions input-output, the carbon footprint of residents' consumption in China's eight major regions, and the carbon emissions input-output from various sectors in the eight major regions, respectively, revealing significant regional differences [5–7]. In addition, Liu et al. [8] used the 1997 inter-regional input-output table to analyze the transfer of carbon emissions from the perspective of industry and proposed a CO_2_ emissions reduction model for industrial structure adjustment. The calculation demonstrated that structural adjustment of energy consumption of electric power industry and improving the efficiency of energy utilization in heavy industry were effective means to reduce carbon emissions [8]. Su and Ang [9, 10] explored the spatial agglomeration of carbon emissions transfer by calculating the carbon emissions transfer between major regions in China in 2002 and proposed to optimize interregional carbon emissions transfer from the mechanism level [9, 10]. Sun et al. [11] calculated carbon transfers between Indian economic sectors from 1995 to 2009 based on the SRIO model. The study found that CON (construction) transfers the largest amount, and EGW (electricity, gas, water supply) emissions account for 60% of CO_2_ in the secondary industry [11]. Based on the MRIO model, Chen et al. [12] independently compiled China's inter-regional input-output table for 2012, calculated the amount of inter-provincial carbon emissions transfer, and analyzed the inter-provincial carbon equity from the perspective of carbon Gini coefficient [12]. Wang and Hu [13] used an inter-regional bilateral trade carbon transfer measurement model to measure the carbon emissions caused by interprovincial demand and interprovincial exports in China in 2007, 2010, and 2012. The study shows that cooperation and diffusion of emission reduction technologies can be effective suppress interprovincial carbon emissions transfer [13]. Han et al. [14] explored the flow of carbon emissions in China's provinces and other economies from a multi-regional perspective based on Nested IOA [14]. Based on the MRIO model, Wang et al. [15] analyzed the carbon-neutrality-oriented carbon emissions reduction model by studying the consumption-based carbon emissions and carbon transfer at the provincial and industry levels in China [15]. Liu et al. [16] deduced the calculation method of the value chain embodied carbon transfer based on the input-output table balance formula, and found that China's inter-regional value chain embodied carbon transfer and its net value both showed an increasing trend [16].

Regarding the decomposition of the influencing factors of carbon emissions transfer, the popular academic methods include the structural decomposition and the logarithmic mean Divisia index (LMDI) decomposition, both of which have been widely used in various research scenarios. Based on the input-output model, Qian and Yang [17], as well as Jiang [18], employed the structural decomposition method to decompose carbon emissions embodied in international trade between East Asia and the BRICS, respectively [17, 18]. Guo [19], Du and Sun [20] and Huang et al. [21] applied the LMDI method to decompose the total carbon emissions, export embodied carbon emissions, and carbon emissions embodied in inter-provincial trade [19–21].

Existing studies have adequately applied the MRIO model to calculate the international and domestic carbon emissions transfer, used different methods to decompose the influencing factors, and analyzed interprovincial carbon equity from various perspectives and put forward corresponding proposals of carbon emissions reduction. On these bases, this study used a MRIO model to calculate the carbon emissions transfer between provinces based on the three-year data of 2012, 2015, and 2017, analyzed the transfer paths of inter-regional carbon emissions through social network, and then decomposed the factors affecting the net carbon emissions transfer at the provincial level using the LMDI method. We aimed to provide a basis for the construction of a coordinated governance mechanism for regional carbon emissions and emissions reduction plans to promote the achievement of carbon peak and carbon neutrality.

## 3. Interprovincial Carbon Emissions Transfer

Different provinces and cities in China have different resource endowments and natural environments. When designing emissions reduction plans, it will be not all-inclusive to consider only the differences in industrial structure between provinces and cities caused by different resource endowments. During the implementation of the emission reduction plans, because interprovincial trade will drive the transfer of interprovincial carbon emissions, with no restriction, some regions will purchase high-carbon emission products from other regions to replace local production for the purpose of carbon reduction, sabotaging overall emissions reduction. Due to differences in technological levels between regions, there may even be reduced partial emissions with increased overall emissions, the so called inter-regional carbon leakage. For that reason, we attempted to provide a basis for the coordinated governance mechanism of regional carbon emissions by calculating and analyzing the current interprovincial carbon emissions transfer, carbon transfer network, and carbon transfer path.

### 3.1. Interprovincial Carbon Emissions Transfer Calculation Model

In the present study, a MRIO model was adopted to calculate the carbon emissions from both the consumers' perspective and the producers' perspective and the inter-region carbon emissions transfers between 31 provinces and cities across China.

Assuming that in the MRIO model there are totally *n* provinces and cities and *m* industries in each province/city, the MRIO model has the following equation:

(1) $$\begin{array}{c} \left[\begin{array}{c} X_{1}^{1}\\ X_{2}^{1}\\ \vdots \\ X_{m}^{n} \end{array}\right]=\left[\begin{array}{c} X_{1,1}^{1,1}+X_{1,2}^{1,1}+\cdots +X_{1,m}^{1,n}\\ X_{2,1}^{1,1}+X_{2,2}^{1,1}\cdots +X_{2,m}^{1,n}\\ \vdots \\ X_{m,1}^{n,1}+X_{m,2}^{n,1}+\cdots +X_{m,m}^{n,n} \end{array}\right]+\left[\begin{array}{c} Y_{1}^{1,1}+Y_{1}^{1,2}+\cdots +Y_{1}^{1,n}\\ Y_{2}^{1,1}+Y_{2}^{1,2}+\cdots +Y_{2}^{1,n}\\ \vdots \\ Y_{m}^{n,1}+Y_{m}^{n,2}+\cdots +Y_{m}^{n,n} \end{array}\right]+\left[\begin{array}{c} {\text{EX}}_{1}^{1}\\ {\text{EX}}_{2}^{1}\\ \vdots \\ {\text{EX}}_{m}^{n} \end{array}\right], \end{array}$$

where *X*_*i*_^*I*^ represents the total output of the industry *i* of province *I*, *X*_*i*,*j*_^*I*,*J*^ represents the intermediate input of the industry *i* of province *I* to the industry *j* of province *J*, *Y*_*i*_^*I*,*J*^ represents the final use of the industry *i* of province *I* by province *J*, and EX_*i*_^*I*^ represents the output of the industry *i* of province *I*. Transform the intermediate input to get:

(2) $$\begin{array}{c} \left[\begin{array}{c} X_{1}^{1}\\ X_{2}^{1}\\ \vdots \\ X_{m}^{n} \end{array}\right]=\left[\begin{array}{cccc} A_{1,1}^{1,1} & A_{1,2}^{1,1} & \cdots & A_{1,m}^{1,n}\\ A_{2,1}^{1,1} & A_{2,2}^{1,1} & \cdots & A_{2,m}^{1,n}\\ \vdots & \vdots & \ddots & \vdots \\ A_{m,1}^{n,1} & A_{m,2}^{n,1} & \cdots & A_{m,m}^{n,n} \end{array}\right]\left[\begin{array}{c} X_{1}^{1}\\ X_{2}^{1}\\ \vdots \\ X_{m}^{n} \end{array}\right]+\left[\begin{array}{c} Y_{1}^{1,1}+Y_{1}^{1,2}\cdots +Y_{1}^{1,n}\\ Y_{2}^{1,1}+Y_{2}^{1,2}\cdots +Y_{2}^{1,n}\\ \vdots \\ Y_{m}^{n,1}+Y_{m}^{n,2}\cdots +Y_{m}^{n,n} \end{array}\right]+\left[\begin{array}{c} {\text{EX}}_{1}^{1}\\ {\text{EX}}_{2}^{1}\\ \vdots \\ {\text{EX}}_{m}^{n} \end{array}\right], \end{array}$$

where *A*_*i*,*j*_^*I*,*J*^=*X*_*i*,*j*_^*I*,*J*^/∑_*k*=1_^*n*^∑_*l*=1_^*m*^*X*_*l*,*j*_^*k*,*J*^ represents the products and services of the industry *i* of province *I* directly consumed by per unit of output of the industry *j* of province *J*.

The matrix form of equation (2) is

(3) $$\begin{array}{c} X=\text{AX}+Y+\text{EX,} \end{array}$$

where *X* is the total output vector (*mn* × 1), *A* is the direct consumption coefficient matrix (*mn* × *mn*), *Y* is the final use vector (*mn* × 1), and EX is the export vector (*mn* × 1).

Transform equation (3) to get

(4) $$\begin{array}{c} X={\left(I-A\right)}^{-1}\left(Y+\text{EX}\right). \end{array}$$

Let *B*=(*I* − *A*)^−1^, where *B* is the total input consumption coefficient matrix and and the element *B*_*i*, *j*_^*I*, *J*^ represents the intermediate products and final products provided by the industry *i* of province *I* to meet the per unit of final use in the industry *j* of province *J*.

Following the Intergovernmental Panel on Climate Change (IPCC) guidelines, this study calculates the carbon emissions of each industry in each province/city using the following equation:

(5) $$\begin{array}{c} C_{i}^{I}=\underset{k}{\sum }{\text{CE}}_{ik}^{I}\\ =\underset{k}{\sum }{\text{AD}}_{ik}^{I}\times {\text{NCV}}_{k}^{I}\times {\text{CC}}_{i}^{I}\times O_{ik}^{I}, \end{array}$$

where *C*_*i*_^*I*^ is the total carbon emissions of the industry *i* of province *I*, CE_*ik*_^*I*^ is the carbon emitted by industry *i* of province *I* consuming energy *k*, AD_*ik*_^*I*^ represents the total amount of energy *k* consumed by the industry *i* of province *I*, NCV_*k*_ represents the average low calorific value of energy *k*, CC_*k*_ is the carbon emissions per unit of heat, and *O*_*ik*_^*I*^ is the oxidation rate.

Then the total carbon emissions caused by final consumption is

(6) $$\begin{array}{c} \text{CE}=\hat{E}{\left(I-A\right)}^{-1}Y, \end{array}$$

where $\hat{E}$ is the carbon emissions intensity coefficient matrix (*E*_*i*_^*I*^=*C*_*i*_^*I*^/*X*_*i*_^*I*^):

(7) $$\begin{array}{c} \hat{E}=\left(\begin{array}{ccccc} E_{1}^{1} & 0 & \cdots & \cdots & 0\\ 0 & \ddots & \vdots & \vdots & 0\\ \vdots & \cdots & E_{i}^{I} & \vdots & \vdots \\ \vdots & \cdots & \cdots & \ddots & 0\\ 0 & 0 & \cdots & 0 & E_{m}^{n} \end{array}\right). \end{array}$$

From the producers' perspective, the carbon emissions CE_*p*_^*I*^ represent the CO_2_ emissions produced by the production activity in province *I*:

(8) $$\begin{array}{c} {\text{CE}}_{p}^{I}=\overset{m}{\underset{K=1}{\sum }}{\hat{E}}^{I}{\left(I-A\right)}^{-1}Y^{K}. \end{array}$$

From the consumers' perspective, the carbon emissions CE_*c*_^*I*^ represents the CO_2_ emissions from production activities in all provinces caused by province *I*'s demand for products and services:

(9) $$\begin{array}{c} {\text{CE}}_{c}^{I}=\overset{m}{\underset{K=1}{\sum }}{\hat{E}}^{K}{\left(I-A\right)}^{-1}Y^{I}. \end{array}$$

Interprovincial carbon emissions transfer originates from interprovincial trade. That is, in order to meet the needs of other provinces and cities for products and services, one province or city needs to conduct a production activity. Then the amount of carbon emissions transfer CT^*I*, *J*^ is represented by the carbon emissions generated during the production process. It means that the carbon emissions of various industries in province *I* caused by the final use by province *J* is the carbon emissions transfer from province *J* to province *I*:

(10) $$\begin{array}{c} {\text{CT}}^{I,J}={\hat{E}}^{I}{\left(I-A\right)}^{-1}Y^{J}. \end{array}$$

### 3.2. Carbon Emissions Results from Different Perspectives

This study adopted the interregional input-output tables of 42 sectors in 31 provinces, municipalities, and autonomous regions in China in 2012, 2015, and 2017. According to the method of Chen et al. [12], the 42 sectors in the input-output tables were merged into 16 sectors. The energy consumption by each sector, each region in each year was from the official data released by CEADs (https://www.ceads.net).The results are shown in Table 1.

In 2012, 2015, and 2017, the highest total carbon emissions from the consumers' perspective were coastal provinces with large populations and high consumption levels, such as Shandong (566 million tons per year) and Guangdong (528 million tons per year). The lower ones were in regions with relatively small populations and low consumption levels including Tibet (15 million tons per year) and Qinghai (41 million tons per year). The primary reason is the large differences in the total population and consumption levels between regions. A large population and a high consumption level make the total final use large in that region, leading to high-carbon emissions from the consumers' perspective. It is the opposite way in sparsely populated areas with low consumption levels.

With respect to the time trend, benefit from the increase in consumption levels by economic development during the period, the total carbon emissions from the consumers' perspective in populous regions such as Guangdong, Jiangsu, and Henan were all rising. While regions such as Beijing, Shanghai, and Tianjin which already have high consumption levels demonstrated an overall decreasing pattern from the perspective of consumers. It indicated that economic development in areas with high consumption levels has brought about a low-carbon consumption structure.

From the perspective of the producers, the high total carbon emissions were in regions like Shandong (645 million tons per year), Hebei (556 million tons per year), and Shanxi (379 million tons per year). The industrial structure in these regions preferred high polluting industries such as steel and energy. The advantageous industries in Shandong include three high-polluting and high-energy-consuming industries, energy processing (petroleum and coal), nonferrous extractive metallurgy, and chemical raw material production, which resulted in much higher carbon emissions than other regions. The lower total carbon emissions from the perspective of producers were regions with advantages in the tertiary industry, such as Beijing (58 million tons per year), Hainan (35 million tons per year), and Tibet (0.05 million tons per year).

Regarding the time trend, the total carbon emissions were relatively stable from the perspective of producers, different from those of the perspective of consumers. Except for Inner Mongolia and Xinjiang whose changes were relatively big, the changes in total carbon emissions were small in other provinces/cities. The primary reason is that their industrial structure is stable compared to the consumption level; therefore, the changes in total carbon emissions caused by industrial structure were small.

### 3.3. Total Net Carbon Emissions Transfer

According to the principle of “the consumers assume responsibility,” the net carbon emissions transfer was calculated by subtracting the producers' total carbon emissions from the consumers' total carbon emissions, that is, equation (9) minus equation (8).

The regions with high annual net carbon emissions transfer-out in 2012, 2015 and 2017 were provinces/cities with large populations and high consumption levels such as Guangdong, Zhejiang, and Beijing. Their industrial structure could not support their own demand, so they purchased products from other provinces/cities and transferred the CO_2_ that should be emitted in region for producing these products to other regions. The regions with high annual net carbon emissions transfer-in were regions with an industry structure preferring energy and heavy-industry, such as Inner Mongolia, Hebei, and Shanxi. On the one hand, the energy industry, as an important part of production and life, participates in the production process of other industries in the region. On the other hand, the region's own demand is far from releasing its production capacity. While a large number of products were sold by these regions, the CO_2_ that should be emitted by other provinces/cities for producing these products was transferred in. With respect to the time trend, provinces/cities with negative annual net carbon emissions transfer have greater fluctuations compared to those with positive annual net carbon transfer. Especially the fluctuations in Chongqing, Yunnan, Shanghai, and Henan were big. While Shanghai and Henan even changed the direction of net carbon transfer.

**Figure 1:**
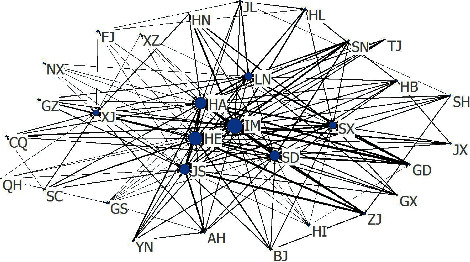
Interprovincial carbon emissions transfer social network (2017).

By equation (10), we can get the interprovincial carbon emission transfer matrix, but it is difficult to display due to the large amount of data. To visualize the carbon emissions transfer relationship clearly between provinces/cities, the amount of carbon transfer from one province/city to the other provinces/cities was sorted by the proportion of the total transfer amount, and the top 60% was taken to generate the interprovincial carbon emissions transfer relationship matrix. Then the social network was drawn with arrows pointed the direction of CO_2_ transfer (see Figure 1).

In general, the interprovincial carbon emissions transfer social network exhibited a star-like shape. The five provinces of Inner Mongolia (IM), Hebei (HB), Shanxi (SX), Liaoning (LN), and Shandong (SD) with high net carbon transfer-in not only received most transfer-out from Guangdong (GD), Zhejiang (ZJ), Beijing (BJ), Chongqing (CQ), Shanghai (SH), and Tianjin (TJ), the regions with high net carbon transfer-out but also served as the main transfer-out places of carbon emissions from other provinces/cities. They worked as the center of the interprovincial carbon emissions transfer network and were also the dominant regions from the perspective of producers. From the perspective of coordinated governance of regional carbon emissions, the impact of the emissions reduction policy on the central provinces could quickly spread through the transfer network, conducive to timely adjustment of policy based on actual effects.

Although the net carbon transfer-in Henan was not high in 2015, and the net carbon emissions were transferred-out in 2017, it is still close to the center of the carbon emissions transfer network. It is because that the carbon emissions transferred to the “sub-center” Henan were far less than the amount transferred to the “central” provinces, and the carbon emissions transferred from Henan to other provinces were high too. Similarly, Jiangsu had a net carbon transfer-out of only 0.02 million tons in 2017, whereas it was still close to the center of the network. The main reason is also that provinces such as Inner Mongolia, Shanxi, and Liaoning received most of the carbon emissions transfer, while carbon emissions transferred from Jiangsu's to other provinces was high too.

## 4. Decomposition of Factors Affecting Net Carbon Emissions Transfer

To support the construction of a coordinated governance mechanism for regional carbon emissions, it is not only necessary to grasp the current interprovincial carbon emissions transfer and carbon transfer network but also need to fully understand the continuous and systemic influencing factors of net carbon emissions transfer. Thus, this article adopted the LMDI method to decompose the influencing factors of the net carbon emissions transfer at the provincial level.

### 4.1. Establishment of the LMDI Model

In order to explore the key factors affecting the net carbon emission transfer in each province, the contribution of various factors to the carbon emission transfer was evaluated. Since this study focused on the impact of carbon emissions intensity, output structure, demand-output ratio, and total demand on carbon emissions, the following equation is used for analysis:

(11) $$\begin{array}{c} C_{ex}^{I}=\overset{m}{\underset{J=1}{\sum }}\overset{n}{\underset{j=1}{\sum }}\left(\frac{C_{j}^{\left(I,\;J\right)}}{X_{j}^{\left(I,J\right)}}\right)\left(\frac{X_{j}^{\left(I,\;J\right)}}{X^{\left(I,J\right)}}\right)\left(\frac{X^{\left(I,J\right)}}{Y_{T}^{I}}\right)Y_{T}^{I}, \end{array}$$

where *E*_*ex*_^(*I*, *J*)*j*^=*C*_*j*_^(*I*, *J*)^/*X*_*j*_^(*I*, *J*)^, representing the carbon emissions transferred per unit of output from province *I* to industry *j* of province *J*; ST_*ex*_^(*I*, *J*)*j*^=*X*_*j*_^(*I*, *J*)^/*X*^(*I*, *J*)^, representing the industry structure of production activities of province *J* to meet the needs of province *I*; *F*_*ex*_^(*I*, *J*)*j*^=*X*^(*I*, *J*)^/*Y*_*T*_^*I*^, representing the output of province *J* caused by per unit of final demand of province *I*; and *Y*_*ex*_^(*I*, *J*)^=*Y*_*T*_^*I*^, representing the final demand scale of province *I*.

(12) $$\begin{array}{c} C_{im}^{I}=\overset{m}{\underset{J=1}{\sum }}\overset{n}{\underset{j=1}{\sum }}\frac{C_{j}^{\left(J,I\right)}}{X_{j}^{\left(J,I\right)}}\frac{X_{j}^{\left(J,I\right)}}{X^{\left(J,I\right)}}\frac{X^{\left(J,I\right)}}{Y_{T}^{J}}Y_{T}^{J}, \end{array}$$

where *E*_*im*_^(*I*,  *J*)*j*^=*C*_*j*_^(*J*,  *I*)^/*X*_*j*_^(*J*,  *I*)^, representing the carbon emissions transferred per unit of output from industry *j* of province *J* to province *I*; ST_*im*_^(*I*,  *J*)*j*^=*X*_*j*_^(*J*,  *I*)^/*X*^(*J*,  *I*)^, representing the industry structure of production activities of province *I* to meet the needs of province *J*; *F*_*im*_^(*I*,  *J*)*j*^=*X*^(*J*,  *I*)^/*Y*_*T*_^*J*^, representing the output of province *I* caused by per unit of final demand of province *J*; and *Y*_*im*_^(*I*, *J*)^=*Y*_*T*_^*J*^, representing the final demand scale of province *J*.

This article employed the LMDI method to decompose the factors that affect the provincial net carbon emissions into technical effect, structural effect, input-output effect, and scale effect:

(13) $$\begin{array}{c} \Delta C^{I}=C_{ex}^{I}-C_{im}^{I}\\ =\Delta C_{E}^{I}+\Delta C_{ST}^{I}+\Delta C_{F}^{I}+\Delta C_{Y}^{I},\\ \Delta C_{E}^{I}=\overset{m}{\underset{J=1}{\sum }}\overset{n}{\underset{j=1}{\sum }}W^{\left(I,J\right)j}\ln\frac{E_{ex}^{\left(I,J\right)j}}{E_{im}^{\left(I,J\right)j}}\cdots \cdots \text{technical effect},\\ \Delta C_{\text{ST}}^{I}=\overset{m}{\underset{J=1}{\sum }}\overset{n}{\underset{j=1}{\sum }}W^{\left(I,J\right)j}\ln\frac{{\text{ST}}_{ex}^{\left(I,J\right)j}}{{\text{ST}}_{im}^{\left(I,J\right)j}}\cdots \cdots \text{structural effect},\\ \Delta C_{F}^{I}=\overset{m}{\underset{J=1}{\sum }}\overset{n}{\underset{j=1}{\sum }}W^{\left(I,J\right)j}\ln\frac{F_{ex}^{\left(I,J\right)j}}{F_{im}^{\left(I,J\right)j}}\cdots \cdots \text{input}-\text{output effect},\\ \Delta C_{Y}^{I}=\overset{m}{\underset{J=1}{\sum }}\overset{n}{\underset{j=1}{\sum }}W^{\left(I,J\right)j}\ln\frac{Y_{ex}^{\left(I,J\right)}}{Y_{im}^{\left(I,J\right)}}\cdots \cdots \text{scale effect}, \end{array}$$

where $W^{\left(I,J\right)j}=\left\{\begin{array}{c} \left(C_{j}^{\left(I,J\right)}-C_{j}^{\left(J,I\right)}\right)/\ln\left(C_{j}^{\left(I,J\right)}-C_{j}^{\left(I,J\right)}\right),\;C_{j}^{\left(I,J\right)}\neq C_{j}^{\left(I,J\right)}\\ C_{j}^{\left(I,J\right)},\;C_{j}^{\left(I,J\right)}=C_{j}^{\left(I,J\right)} \end{array}\right.$.

The technology effect reflects the differences in production technology between provinces in interprovincial trade. The structural effect reflects the contribution of the difference n the final demand structure of each province to the transfer of net carbon emissions. The input-output effect reflects the degree of correlation between the various industries in each province in the production process. The scale effect reflects the contribution of differences in the scale of final demand across provinces to the transfer of net carbon emissions implied by trade.

### 4.2. Analysis of LMDI Decomposition Results

This paper decomposed the annual net carbon transfer into technical effect, structural effect, input-output effect, and scale effect via LMDI method. A positive contribution of an effect indicated that this effect made the regional carbon transfer-out greater than carbon transfer-in, and a negative contribution value indicated a larger carbon transfer-in than carbon transfer-out. The decomposition results are shown in Table 2. Due to the large amount of data, only data of 2017 are displayed.

The provinces/cities with a positive technical effect were concentrated in the economy developed regions such as Beijing, Guangdong, and Zhejiang, as well as the regions whose economic development did not rely on high-polluting and high-emission industries, such as Jiangxi and Yunnan. The main reasons might be that, the regions such as Beijing and Guangdong, have advanced carbon emission technology with developed economy compared to their trading regions, resulting smaller amount of carbon emissions of the corresponding equivalent commodities. Therefore, the carbon transfer-in caused by production was less than the carbon transfer-out caused by consumption. Provinces and cities that do not rely on high-polluting and high-emission industry for economic development such as Jiangxi and Yunnan had advantages in carbon emissions intensity when trading with regions with a large proportion of heavy industry, leading to net carbon emissions transfer-out. On this basis, during the coordinated governance of regional carbon emissions, we could encourage technology exchanges between regions with positive technical effects and regions with negative technical effect to speed up the spread of current low-carbon production technologies. In addition, we could strengthen support for technological innovation in regional with negative technical effect to guide the production technology toward low-carbon style.

The regions with positive structural effect included 16 provinces and cities, such as Jiangsu, Chongqing, Shanghai, and Tianjin. The main reason is that the provinces and cities, such as Jiangsu, Chongqing, and Shanghai, when trading with other regions, gained output in industries with low-carbon emissions such as transportation and storage and construction in their own regions. While the provinces such as Inner Mongolia, Shanxi, and Ningxia, gained output in industries with high-carbon emissions such as coal mining and dressing, metal smelting and pressing, and power and heat supply. Therefore, the regions with positive structural effect would have a smaller carbon emissions transfer-in from production than the carbon emissions transfer-out from consumption, thus a net carbon transfer-out. Whereas the regions with negative structural effect would have a larger carbon transfer-in than transfer-out, thus a new carbon transfer-in. From the perspective of collaborative governance, it is necessary to fully consider the differences in resource endowments between provinces and cities, to guide the industrial structure of each region to give full play to its own advantages, and to rationally lead the transfer of high-polluting and high-emission industries between regions.

The regions with positive input-output effect were mainly distributed in areas with relatively immature industrial chains and weak economies such as the northwest and southwest. While regions with negative input-output effect were mainly distributed in the eastern and northern coastal areas, where the industrial chains were more mature and the input-output relationship between industries was more complex. When meeting the needs of other provinces, the total output increase of all industries brought by the equivalent demand from other provinces will be higher than that of regions with weaker inter-industry linkages. It is likely to cause larger carbon transfer-in from production than carbon transfer-out from consumption, thus the net carbon transfer-in. The input-output effect was the dominant factor of net carbon transfer. Therefore, in the overall governance process, it is necessary to rationally consider the economic benefits of the increased production of various industries brought about by the interprovincial trade demand and the carbon emissions transfer that comes along, so as to improve the interprovincial industrial chain. Optimizing the industrial structure and leading the input-output relationship between industries toward low-carbon can be an effective practical direction to promote the coordinated governance of regional carbon emissions.

The regions with positive scale effect were mainly distributed in developed coastal provinces. They have large populations and high consumption levels, and the corresponding final consumption scales were larger than other provinces. Therefore, the scale of consumption would be greater than the scale of production in interprovincial trade, and the carbon emissions transfer-out from consumption would be larger than transfer-in from production, resulting in a net carbon transfer-out. From the perspective of coordinated governance of regional carbon emissions, it is necessary to build a reasonable horizontal inter-provincial carbon compensation mechanism, so that high-consumption regions could assume the carbon emission responsibility of consumed goods, and build a bridge of coordinated carbon governance between carbon transfer-out and transfer-in provinces and cities.

In terms of time trend, among the provinces with large changes in carbon emissions transfers in 2012, 2015, and 2017, the changes in total effect in Inner Mongolia, Liaoning, Jilin, Heilongjiang, Hunan, and Zhejiang were mainly due to the same direction changes of scale effect. The decline in total effect in Fujian was primarily due to the reduction in input-output effect. While for provinces and cities with drastic changes in the amount of carbon emissions transfer, the changes in total effect in Shanghai, Jiangsu, and Henan were mainly caused by the same direction changes of scale effect. It can be seen that the scale effect was the dominant factor causing changes in carbon emissions transfer. Presumably, it is because that the final consumption scale responded more quickly to changes in environmental factors such as consumers' preferences and industry policies than the industrial structure, technological level, and industry input-output relationships.

## 5. Conclusion and Suggestion

This study constructed an interregional input-output model for 31 provinces and cities in China to calculate and analyze the carbon emissions transfer between provinces and cities, carbon emissions from the perspective of producers and from the perspective of consumers. Furthermore, this study conducted influencing factor decomposition for net carbon emissions transfer using the LMDI method. The following conclusions are drawn:

1. High-carbon emission areas from the consumers' perspective are mainly distributed in populous provinces and cities such as Shandong, Guangdong, Jiangsu, and Henan, and the emissions are rising. While in regions with high consumption levels such as Beijing, Shanghai, and Tianjin, the carbon emissions are declining from the consumers' perspective. High-carbon emission areas from the producers' perspective are mainly distributed in regions with industrial structure preference toward high-polluting industries like steel and energy, including Shandong, Hebei, and Shanxi. And the carbon emissions the producers' perspective fluctuated little over time and are relatively stable.
2. The interprovincial carbon emissions transfer network is star-like. The five provinces, Inner Mongolia, Hebei, Shanxi, Liaoning, and Shandong, with high net carbon transfer-in are the primary transfer-out places of carbon emissions from other provinces, and they are located in the center of the carbon emissions transfer network.
3. The input-output effect is the dominant one that affects the net carbon transfer, indicating that the net carbon transfer is primarily affected by the completeness degree of the industrial chain. In the eastern and northern coastal areas, the industrial chain is relatively complete, and the input-output relationship between industries is more complicated. Their increases in total industrial output caused by the same demand are higher than that of other regions. Therefore, the carbon transfer-in from production is greater than the carbon transfer-out from consumption, resulting a net carbon transfer-in. The scale effect is the primary factor determining the carbon emissions transfer from 2012 to 2017. The reason for that is that compared with the industrial structure, technological level, and industry input-output relationships, the final consumption scale responds more quickly to changes in environmental factors including consumers' preferences and industry policies.

Based on the above-mentioned empirical results and research conclusions, the following suggestions are made. First, we should strengthen support for low-carbon industries and low-carbon technologies. On the one hand, to provide more support for low-carbon industries such as clean energy to improve energy efficiency and expedite the low-carbon shift in industrial structure. On the other hand, to encourage low-carbon technological innovation and lead high-polluting and high-emission industries to reduce pollution and emissions. At the same time, to promote low-carbon consumption and low-carbon lifestyle and guide the low-carbon shift in consumption concept. We should take full advantage of the relationship between the center regions with other regions in the interprovincial carbon emissions transfer network, prioritize the industrial upgrading and transformation of the metal and nonmetal processing and manufacturing industry in center regions, and eliminate outdated production capacity with high pollution and high emissions. Then, we should improve the responsibility mechanism for interprovincial carbon transfer. We should fully review the differences in economic development between provinces, comprehensively consider the producers' principle and consumers' principle, and rationally allocate carbon reduction responsibility in major high-carbon production regions and major high-carbon consumption regions. Last but not least, we should think about the differences in resource endowments of various provinces and cities, improve the industrial chain, and promote the low-carbon shift of input-output structure of inter-provincial industries.

## Figures and Tables

**Table 1:** Carbon emissions results from different perspectives.

Year	Consumers' perspective			Producers' perspective		
	2012	2015	2017	2012	2015	2017
Beijing	209.0	170.9	142.7	65.5	59.6	49.4
Tianjin	202.0	199.7	117.4	112.5	112.5	104.1
Hebei	351.9	369.2	476.1	536.6	556.0	576.5
Shanxi	227.8	209.6	241.6	378.1	355.2	406.4
Inner Mongolia	291.0	219.1	263.9	455.9	463.6	549.2
Liaoning	302.5	304.7	240.1	356.3	371.0	371.4
Jilin	223.0	209.4	175.7	205.3	185.2	179.6
Heilongjiang	261.8	223.8	239.3	222.6	228.6	232.5
Shanghai	277.4	161.7	122.9	123.0	115.1	132.3
Jiangsu	409.9	431.7	517.5	436.5	504.5	515.9
Zhejiang	320.1	420.1	435.8	252.7	264.0	258.3
Anhui	185.0	257.9	246.7	258.1	289.6	310.2
Fujian	191.2	148.2	135.4	165.8	169.7	178.9
Jiangxi	128.0	184.3	196.9	128.7	166.7	182.0
Shandong	566.4	602.0	532.1	640.0	662.0	635.6
Henan	356.9	426.0	517.8	419.8	435.1	407.7
Hubei	349.5	379.0	316.4	310.7	269.5	277.9
Hunan	241.4	291.7	351.7	237.0	239.5	265.4
Guangdong	559.9	438.0	586.0	298.6	294.5	345.0
Guangxi	203.2	202.3	175.7	173.4	171.6	186.0
Hainan	50.1	53.9	36.0	32.4	36.3	37.1
Chongqing	159.0	291.6	197.1	138.8	131.4	124.4
Sichuan	233.1	269.7	289.6	258.7	272.8	263.8
Guizhou	131.0	161.3	199.5	182.4	200.1	209.5
Yunnan	200.2	244.7	278.1	176.5	150.4	169.0
Tibet	12.4	8.5	24.6	4.0	4.4	6.6
Shaanxi	220.6	238.1	270.1	191.8	227.2	217.6
Gansu	90.7	105.9	96.5	121.5	132.7	126.1
Qinhai	34.6	36.8	54.5	34.0	42.1	46.9
Ningxia	50.4	56.0	99.4	108.9	119.1	145.6
Xinjiang	181.3	191.5	273.7	195.2	277.1	339.7

**Table 2:** LMDI decomposition results in 2017.

Province	Total effect	Total effect	Total effect	Technical effect	Structural effect	Input-output effect	Scale effect
Year	2012	2015	2017	2017			
Beijing	1.44	1.11	0.93	1.30	−−0.17	−0.15	−0.05
Tianjin	0.90	0.87	0.13	0.35	−0.03	0.10	−0.29
Hebei	−1.85	−1.87	−1.00	−0.63	0.15	−0.80	0.27
Shanxi	−1.50	−1.46	−1.65	−0.79	−0.92	0.85	−0.78
Inner Mongolia	−1.65	−2.44	−2.85	−1.55	−1.34	1.09	−1.05
Liaoning	−0.54	−0.66	−1.31	−0.58	−0.55	0.14	−0.32
Jilin	0.18	0.24	−0.04	−0.17	0.39	0.35	−0.61
Heilongjiang	0.39	−0.05	0.07	−0.68	0.18	1.00	−0.43
Shanghai	1.54	0.47	−0.09	0.22	0.37	−0.45	−0.24
Jiangsu	−0.27	−0.73	0.02	0.31	0.76	−3.05	2.00
Zhejiang	0.67	1.56	1.78	0.96	0.21	−0.34	0.93
Anhui	−0.73	−0.32	−0.64	0.04	0.20	−0.63	−0.24
Fujian	0.25	−0.22	−0.43	0.23	−0.33	−0.33	−0.01
Jiangxi	−0.01	0.18	0.15	0.25	0.19	0.13	−0.41
Shandong	−0.74	−0.60	−1.04	0.04	−0.44	−1.99	1.35
Henan	−0.63	−0.09	1.10	0.70	0.62	−1.76	1.55
Hubei	0.39	1.09	0.38	0.00	0.02	0.17	0.20
Hunan	0.04	0.52	0.86	0.15	0.59	−0.09	0.22
Guangdong	2.61	1.43	2.41	1.28	1.31	−2.10	1.92
Guangxi	0.30	0.31	−0.10	−0.11	−0.03	0.34	−0.30
Hainan	0.18	0.18	−0.01	−0.02	−0.01	0.44	−0.42
Chongqing	0.20	1.60	0.73	0.13	0.70	0.28	−0.39
Sichuan	−0.26	−0.03	0.26	0.23	0.04	−0.20	0.19
Guizhou	−0.51	−0.39	−0.10	−0.07	−0.34	0.86	−0.55
Yunnan	0.24	0.94	1.09	0.06	0.04	1.07	−0.08
Tibet	0.08	0.04	0.18	0.09	0.05	0.11	−0.07
Shaanxi	0.29	0.11	0.53	−0.16	0.44	0.57	−0.32
Gansu	−0.31	−0.27	−0.30	−0.10	−0.31	0.66	−0.54
Qinghai	0.01	−0.05	0.08	0.00	−0.06	0.30	−0.18
Ningxia	−0.58	−0.63	−0.46	−0.42	−0.76	1.52	−0.80
Xinjiang	−0.14	−0.86	−0.66	−1.05	−0.93	1.89	−0.57

## Data Availability

Energy consumption by each sector and each region in each year was taken from the official data released by CEADs (https://www.ceads.net).
